# Commentary: The Nomenclature of Human White Matter Association Pathways: Proposal for a Systematic Taxonomic Anatomical Classification

**DOI:** 10.3389/fnana.2019.00061

**Published:** 2019-06-11

**Authors:** Sandip S. Panesar, Juan Fernandez-Miranda

**Affiliations:** Department of Neurosurgery, Stanford University, Stanford, CA, United States

**Keywords:** tractography, association tracts, white matter, neuroanatomy, arcuate fasciculus

The anatomical study of mammalian white matter structure and arrangement has evolved over the past two centuries. There have been three major revolutions facilitating neuroanatomical study of white matter: Early development of post-mortem preparation techniques like Klingler's (Ludwig and Klingler, 1956) to readily visualize the gross orientation of larger white fibers in dissection specimens; introduction of neurochemical tracing techniques to permit cortical-connectivity analysis in non-human primates in the second half of the twentieth century (Lanciego and Wouterlood, 2011); and most recently, development of *in vivo* diffusion magnetic resonance (MR) tractography in the 1990's, which has grown to become the premier white matter research technique. Unfortunately, and for varying reasons, the anatomical classification and nomenclature of white matter architecture, particularly of the association fasciculi, has been subject to considerable heterogeneity, conflicting theories, and disagreements between researchers. Nevertheless, attempts have been made to unify the classification and nomenclature of the human white matter fasciculi.

Recently, Mandonnet et al. (2018) proposed a global classification for the long-range human association fasciculi on a hierarchical basis, using the insular sulcus as a demarcating boundary of larger dorsal and ventral systems (Mandonnet et al., 2018). Each system contained particular, well-known bundles interconnecting cortical areas, which received novel numerical classification e.g., the arcuate fasciculus is known as the superior longitudinal system IV. Though this attempt to unify the anatomy is commendable, we believe, however, that their proposal potentially adds more confusion to the current scenario. From our perspective, the major shortcoming of this nomenclature is that it does not adequately dispel the archaic, conflicting notions of white matter anatomy compounded over the years, but rather “paints over” them while leaving the fundamental controversies unaddressed, or possibly causing greater confusion.

Though it is unfeasible to discuss every association tract discussed in the current classification, we use our own recently published data regarding several relevant tracts (Fernández-Miranda et al., 2015; Wang et al., 2016; Panesar et al., 2017, 2018a,b) to argue our point: The superior longitudinal fascicle (SLF) was first divided into 4 subsegments (SLF I to IV) based on primate neurochemical tracer data (Petrides and Pandya, 1984), and later reinforced by a diffusion tensor imaging (DTI) study (Makris et al., 2005). These initial studies included the arcuate fasciculus (AF) as the “SLF-IV” or “perisylvian-SLF,” a proposal later propagated by others (Catani et al., 2005; Martino et al., 2013). The present authors propose the AF to be considered as the “superior longitudinal system (SLS) IV.” In our view, this is problematic: The AF cannot be regarded as a longitudinal tract as it is an “arcuate-shaped” tract; in fact, the AF is a lateral fronto-temporal fascicle with no parietal connections (Fernández-Miranda et al., 2015), while the SLF is a lateral fronto-parietal fascicle with no temporal connections (Wang et al., 2016). From an anatomical (morphological and topographical) perspective, defining an “arcuate” tract as a “longitudinal” tract is misleading.

Furthermore, the literature generally shows that the AF has strong leftward-lateralization in terms of its subdivisions, volume, and connectivity profiles, while the “superior longitudinal fasciculus proper” (excluding AF) (Thiebaut de Schotten et al., 2011; Wang et al., 2016), is rightward-lateralized in terms of subdivision, connectivity, and volume. Based upon comparisons between human and simian AF morphology, Rilling et al. (2008) proposed the human AF to be evolutionarily differentiated to sub-serve lexical-semantic functionality (Rilling et al., 2008). This view was further elaborated upon and reinforced with our dedicated tractographic and dissection study (Fernández-Miranda et al., 2015). As the structural characteristics of white matter likely reflect evolutionary divergence, underpinned by functional specialization (Glasser and Rilling, 2008; Rilling et al., 2008), this adds further evidence that these anatomo-functionally differentiated should not be grouped together.

In our advanced fiber tractography study of the SLF (Wang et al., 2016), we were unable to find the so-called SLF-I, which in theory travels adjacent to SLF-II to interconnect the superior frontal gyrus with the superior parietal lobule. We did find fibers interconnecting these two regions, but they were traveling medial to the corona radiata in the mesial aspect of the hemisphere. We subsequently proposed these fibers to be part of the cingulum fiber system rather than the SLF. However, multiple authors have continued preserving the inappropriate nomenclature derived from primate studies for no good reason. Our study showed that the SLF can be practically classified in dorsal and ventral components, which correlate with the SLF-II and III, but offer additional anatomical information in their description while adhering to modern anatomical nomenclature systems.

The same issues arise when considering the proposed nomenclature for the “inferior longitudinal system (ILS)” which includes both the inferior fronto-occipital fasciculus (IFOF) and the uncinate fasciculus (UF). According to the authors, the “ILS IV” is synonymous with the UF. Recent tractographic studies have demonstrated a unique, subdivided morphology of the UF (Hau et al., 2016, 2017; Panesar et al., 2017). According to the proposed “ILS” nomenclature, the various subcomponents of the IFOF, in concordance with our previous findings (Panesar et al., 2017) are accounted for, yet the subdivisions of the UF are not (Figure 1).

**Figure 1:**
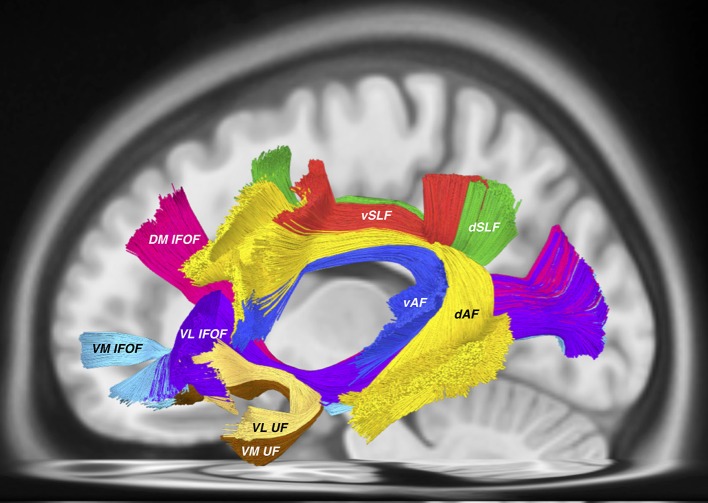
All tractography conducted in DSI studio (http://dsi-studio.labsolver.org) using the HCP 842 atlas (Yeh et al., 2018) as a template. AF tracts were created according to the method described in (Fernández-Miranda et al., 2015), while SLF tracts were created according to the methodology used by Wang et al. (2016). IFOF and UF tracts were created using the method from Panesar et al. (2017). This tractography template represents “averaged” healthy white matter tractographic anatomy of 842 subjects from the Human Connectome Project. Visible in this picture are the dorsal (dAF) and ventral (vAF) AF components, the dorsal (dSLF) and ventral (vSLF) SLF components. Deep and ventral to these tracts are the IFOF consisting of the dorsomedial (DM IFOF), ventromedial (VM IFOF) and ventrolateral (VL) subfascicles. The UF also traverses through the ventral external capsule and is comprised of the ventrolateral (VL UF) and ventromedial (VM UF) sub-fascicles.

Fiber tracts should not be grouped with other fasciculi on the sole basis of spatial proximity, but on the basis of distinct connectivity. In addition, the present proposal carries on with tract sub-classifications that originated from animal studies and were later “validated” with DTI studies. The classifications, from our point of view, are inaccurate and inappropriate for human brain anatomy, especially in light of new tractography findings. We strongly recommend against the use of numerical subsegments (I to IV), and we favor using a topographic classification (dorsal-ventral, medial-lateral), which has a long tradition and is better understood by neuroanatomists as the names themselves provide anatomical information, as opposed to numeric classifications that provide no additional information.

Finally, we highlight technical factors that may potentially confound this classification proposal. At this point, the differences between DTI and more advanced white matter tractography modalities such as high-angular resolution diffusion imaging (HARDI) or generalized Q-sampling imaging (GQI) are well-recognized. In a recent dissection and tractography study into the short vertical association tracts of the posterior hemisphere, we demonstrated that GQI-based tractography could reliably demonstrate the unique spatial separation between the two components within what Mandonnet et al. refer to as the posterior transverse system, and which we refer to as the “temporo-parietal aslant tract” (temporo-parietal course) and vertical occipital fasciculus (occipito-occipital course), respectively (Panesar et al., 2018a). Yeatman et al. (2014) first questioned whether these two fasciculi were indeed separated, a discrete “band of fibers” or whether they appeared unified due to shortcomings of the DTI method. In our study, we demonstrated that the temporo-parietal aslant tract and vertical occipital fasciculus were indeed spatially separated. The band of fibers bridging the two fascicles may be comprised of U-fibers or may be comprised of false continuities from other fasciculi, arising from tensor-based tractography.

In conclusion, we congratulate the authors for the efforts toward a unified classification of the white matter tracts, but at the same time we encourage them and all other experts in the field to consider the points of concern raised here, and to utilize a more practical, anatomically-oriented, and academically-accurate classification of the human fiber tracts.

## Author Contributions

Both authors SP and JF-M contributed equally to the manuscript: JF-M: main idea and editing. SP: writing and figure creation.

### Conflict of Interest Statement

The authors declare that the research was conducted in the absence of any commercial or financial relationships that could be construed as a potential conflict of interest.
